# Possible Eradication of Wild Poliovirus Type 3 — Worldwide, 2012

**Published:** 2014-11-14

**Authors:** Olen M. Kew, Stephen L. Cochi, Hamid S. Jafari, Steven G.F. Wassilak, Eric E. Mast, Ousmane M. Diop, Rudolf H. Tangermann, Gregory L. Armstrong

**Affiliations:** 1 Division of Viral Diseases, National Center for Immunization and Respiratory Diseases, CDC; 2 Global Immunization Division, Center for Global Health, CDC; 3 Polio Eradication Department, World Health Organization

In 1988, the World Health Assembly resolved to eradicate polio worldwide. Since then, four of the six World Health Organization (WHO) regions have been certified as polio-free: the Americas in 1994 (1), the Western Pacific Region in 2000 (2), the European Region in 2002 (3), and the South-East Asia Region in 2014 (4). Currently, nearly 80% of the world’s population lives in areas certified as polio-free. Certification may be considered when ≥3 years have passed since the last isolation of wild poliovirus (WPV) in the presence of sensitive, certification-standard surveillance (1–4).* Although regional eradication has been validated in the European Region and the Western Pacific Region, outbreaks resulting from WPV type 1 (WPV1) imported from known endemic areas were detected and controlled in these regions in 2010 and 2011, respectively (5). The last reported case associated with WPV type 2 (WPV2) was in India in 1999, marking global interruption of WPV2 transmission (6). The completion of polio eradication was declared a programmatic emergency for public health in 2012, and the international spread of WPV1 was declared a public health emergency of international concern in May 2014. The efforts needed to interrupt all indigenous WPV1 transmission are now being focused on the remaining endemic countries: Nigeria, Afghanistan, and Pakistan. WPV type 3 (WPV3) has not been detected in circulation since November 11, 2012. This report summarizes the evidence of possible global interruption of transmission of WPV3, based on surveillance for acute flaccid paralysis (AFP) and environmental surveillance.

## Poliovirus Surveillance

Since the launch of the Global Polio Eradication Initiative in 1988, progress toward eradication has been tracked by detection and investigation of AFP cases and testing of stool specimens for polioviruses by accredited laboratories of the WHO Global Polio Laboratory Network (7). AFP surveillance has been supplemented by environmental surveillance (i.e., testing of sewage samples) in 25 countries, including Pakistan, since 2009, Nigeria since 2011, and Afghanistan since 2013 (7). Environmental surveillance often can detect evidence of WPV infections even in the absence of AFP cases, which is especially important for detection of WPV3, whose case-to-infection ratio (approximately one paralytic case in 1,000 infections) is about one fifth that of WPV1 (approximately one in 200) (8). The quality of AFP surveillance is monitored with performance indicators for detection sensitivity and investigation timeliness (7).

## WPV3 Cases

**FIGURE 1:**
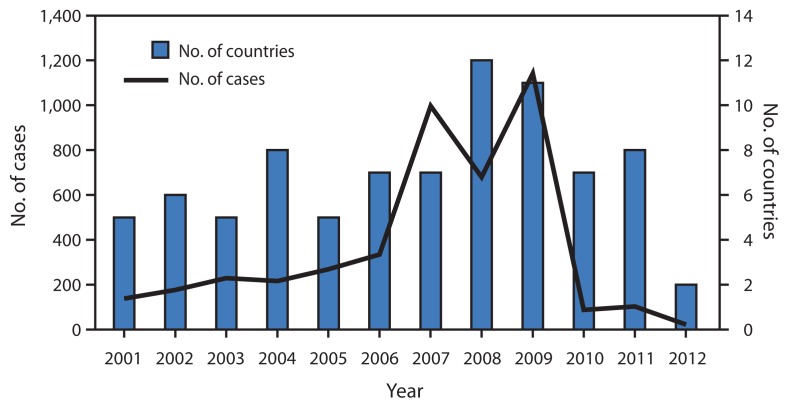
Reported wild poliovirus type 3 (WPV3) cases and countries with reported WPV3 cases, by year — worldwide, 2001–2012

No WPV3 cases have been detected globally since November 2012 (Figure 1). The latest WPV3 in Asia was isolated from a child aged 1 year in the Federally Administered Tribal Area of Pakistan who had onset of AFP on April 18, 2012, and the latest environmental WPV3 isolate in Asia was from a sample collected in Karachi, Pakistan, on October 7, 2010. The latest WPV3 in Africa was isolated from an infant aged 11 months in Yobe, Nigeria, who had onset of paralysis on November 10, 2012, and the latest environmental WPV3 isolate in Africa was from a sample collected in Lagos, Nigeria, on November 11, 2012. The number of countries reporting WPV3 cases changed from five in 2001, to 12 in 2008, to seven in 2010, and to two in 2012 (Figure 1). During 2010–2013, the number of WPV3 isolated globally in stool specimens collected from AFP patients declined from 87 to zero, whereas the number of AFP cases with specimens tested increased from 98,788 in 2010 to 101,701 in 2013.

**FIGURE 2:**
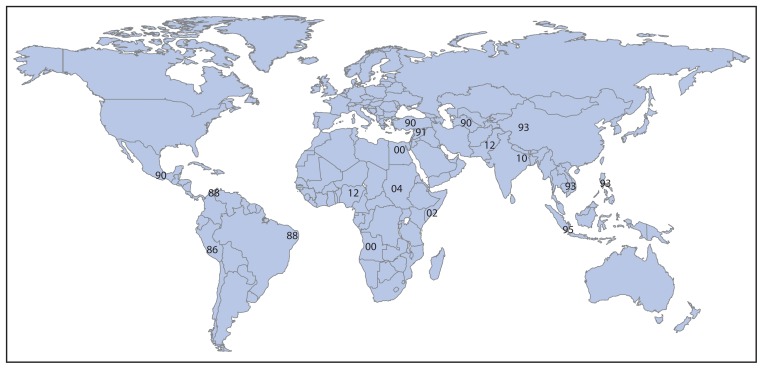
Eradication of wild poliovirus type 3 (WPV3) genotypes — worldwide, 1986–2012* * The two digits represent the last two digits of the year when each WPV3 genotype was last detected.

The genetic diversity of WPV3 isolates has fallen steadily worldwide since 1988. The number of distinct WPV3 genotypes (≥15% nucleotide sequence divergence) detected globally declined from 17 in 1988, to five in 2001, to three in 2010, and to two in 2012 (Figure 2). In Pakistan, WPV3 clusters (approximately 5% nucleotide sequence divergence) within genotypes declined from four in 2010, to one in 2011, and to one in 2012. In Nigeria, the number of WPV3 clusters declined from nine in 2010, to six in 2011, and to two in 2012.

### Discussion

WPV3 has not been detected since November 2012, suggesting that global WPV3 transmission has been interrupted. In regions and areas where the transmission of all three indigenous WPV serotypes has been interrupted, the order of disappearance was first WPV2, then WPV3, and then WPV1. The rapidly declining genetic diversity of WPV3 isolates during the last decade is consistent with progress toward eradication and was observed during a period of improving surveillance performance in Pakistan and Nigeria, the two countries which harbored the last known WPV3 reservoirs (5,7). The possible interruption of WPV3 transmission is a historic milestone for the Global Polio Eradication Initiative and demonstrates that full implementation of the national emergency action plans† in the three remaining polio-endemic countries (Pakistan, Afghanistan, and Nigeria) will also interrupt WPV1 transmission. If validated, the eradication of WPV3 would mark the third time that transmission of a distinct human pathogen (the others are smallpox virus and WPV2) has been interrupted through immunization. The last isolation of WPV2 occurred 15 years ago from a case with onset in October 1999 (6).

In the pre-vaccine era, WPV3 had a worldwide distribution (Figure 2). Although WPV3, for reasons unknown, is less able than WPV1 to spread over wide geographic areas and cause explosive outbreaks, long-range WPV3 exportations and regional outbreaks have occurred (5,9). The substitution of bivalent oral poliovirus vaccine (types 1 and 3) for monovalent oral poliovirus vaccines and trivalent oral poliovirus vaccine (types 1, 2, and 3) in supplementary immunization activities in late 2009 led to the rapid collapse of WPV3 transmission in India the following year, and to the steady decline of WPV3 detection elsewhere (4,5).

Continued sensitive surveillance is needed before the evidence of WPV3 eradication is conclusive, particularly given evidence of remaining limitations of surveillance in Pakistan, Nigeria, and elsewhere (7). The low case-to-infection ratio for WPV3 infections requires higher surveillance sensitivity for detection than that required for WPV1. However, the case-to-infection ratio is lowest for WPV2 (8), and no reappearance has been detected since 1999. High levels of population immunity to poliovirus type 3 should be maintained, both to protect against any residual WPV3 infections and to prevent the emergence and spread of type 3 circulating vaccine-derived polioviruses, a rare event (10).

What is already known on this topic?

Four of the six World Health Organization regions have been certified as polio-free: the Americas in 1994, the Western Pacific Region in 2000, the European Region in 2002, and the South-East Asia Region in 2014. The last detection of wild poliovirus type 2 was in 1999.

What is added by this report?

No type 3 wild poliovirus (WPV3) infections have been detected globally since November 2012, suggesting that transmission might have been interrupted. The number of countries reporting WPV3 isolates declined from seven in 2010 to zero in 2013. During 2010–2013, the number of WPV3 isolated globally in stool specimens collected from patients with acute flaccid paralysis declined from 87 to zero, whereas the number of acute flaccid paralysis cases with specimens tested increased from 98,788 in 2010 to 101,701 in 2013.

What are the implications for public health practice?

Transmission of WPV3 might be interrupted worldwide. Continued sensitive surveillance is needed before the evidence of WPV3 eradication is conclusive, particularly given evidence of limitations of surveillance in Pakistan, Nigeria, and elsewhere.

## Footnotes

* The Global Polio Eradication Initiative sets operational targets for countries in regions with current or recent WPV transmission, both nationally and in each province/state, as ≥2 cases per 100,000 population aged <15 years of nonpolio AFP, and adequate stool specimen collection from ≥80% of AFP cases, in which two specimens are collected ≥24 hours apart, both within 14 days of paralysis onset, shipped on ice or frozen ice packs, and arriving in good condition (without leakage or desiccation) at a WHO-accredited laboratory.

† Emergency action plans have been devised and are revised annually in each endemic country and include all programmatic steps planned to interrupt WPV transmission and respond to outbreaks.
